# Technology for Large-Scale Translation of Clinical Practice Guidelines: A Pilot Study of the Performance of a Hybrid Human and Computer-Assisted Approach

**DOI:** 10.2196/medinform.4450

**Published:** 2015-10-09

**Authors:** Stijn Van de Velde, Lieve Macken, Koen Vanneste, Martine Goossens, Jan Vanschoenbeek, Bert Aertgeerts, Klaar Vanopstal, Robert Vander Stichele, Joost Buysschaert

**Affiliations:** ^1^ EBMPracticeNet Leuven Belgium; ^2^ Belgian Center for Evidence-Based Medicine Leuven Belgium; ^3^ Department of Translation, Interpreting and Communication Ghent University Ghent Belgium; ^4^ IVS Iscientia Keerbergen Belgium; ^5^ Academic Centre for General Practice Katholieke Universiteit Leuven Leuven Belgium; ^6^ Heymans Institute of Pharmacology Ghent University Ghent Belgium

**Keywords:** practice guidelines as topic, translations, technology, education, medical, continuing, evidence-based practice

## Abstract

**Background:**

The construction of EBMPracticeNet, a national electronic point-of-care information platform in Belgium, began in 2011 to optimize quality of care by promoting evidence-based decision making. The project involved, among other tasks, the translation of 940 EBM Guidelines of Duodecim Medical Publications from English into Dutch and French. Considering the scale of the translation process, it was decided to make use of computer-aided translation performed by certificated translators with limited expertise in medical translation. Our consortium used a hybrid approach, involving a human translator supported by a translation memory (using SDL Trados Studio), terminology recognition (using SDL MultiTerm terminology databases) from medical terminology databases, and support from online machine translation. This resulted in a validated translation memory, which is now in use for the translation of new and updated guidelines.

**Objective:**

The objective of this experiment was to evaluate the performance of the hybrid human and computer-assisted approach in comparison with translation unsupported by translation memory and terminology recognition. A comparison was also made with the translation efficiency of an expert medical translator.

**Methods:**

We conducted a pilot study in which two sets of 30 new and 30 updated guidelines were randomized to one of three groups. Comparable guidelines were translated (1) by certificated junior translators without medical specialization using the hybrid method, (2) by an experienced medical translator without this support, and (3) by the same junior translators without the support of the validated translation memory. A medical proofreader who was blinded for the translation procedure, evaluated the translated guidelines for acceptability and adequacy. Translation speed was measured by recording translation and post-editing time. The human translation edit rate was calculated as a metric to evaluate the quality of the translation. A further evaluation was made of translation acceptability and adequacy.

**Results:**

The average number of words per guideline was 1195 and the mean total translation time was 100.2 minutes/1000 words. No meaningful differences were found in the translation speed for new guidelines. The translation of updated guidelines was 59 minutes/1000 words faster (95% CI 2-115; *P*=.044) in the computer-aided group. Revisions due to terminology accounted for one third of the overall revisions by the medical proofreader.

**Conclusions:**

Use of the hybrid human and computer-aided translation by a non-expert translator makes the translation of updates of clinical practice guidelines faster and cheaper because of the benefits of translation memory. For the translation of new guidelines, there was no apparent benefit in comparison with the efficiency of translation unsupported by translation memory (whether by an expert or non-expert translator).

## Introduction

The construction of EBMPracticeNet, a national electronic point-of-care information platform for the Belgian context, was initiated in 2011 to optimize quality of care by promoting evidence-based decision making [1]. The fundamental principle of evidence-based medicine (EBM) is that diagnostic and therapeutic actions must be based on the best available scientific knowledge about possible decisions, supplemented with the clinical expertise of the provider and taking into account the values and preferences of the patient [2]. Evidence-based practice guidelines have been developed to help clinicians keep up to date with current evidence and to support the use of evidence-based medicine in practice. The Institute of Medicine defines guidelines as “Statements that include recommendations intended to optimize patient care that are informed by a systematic review of evidence and an assessment of the benefits and harms of alternative care options.”

Belgian scientific associations of primary care physicians have produced about 50 Belgian clinical practice guidelines of good quality linked to electronic health records through a tool called the Evidence Linker [1]. To be able to provide answers to a broader array of health questions posed by physicians at the point of care, we supplemented the set of guidelines with an international collection of evidence-based point-of-care summaries. Such point-of-care summaries have been defined as Web-based medical compendia specifically designed to deliver predigested, rapidly accessible, comprehensive, periodically updated, and evidence-based information to clinicians [3]. Based on a broad evaluation, we eventually chose to subscribe to the Duodecim Evidence-Based Medicine (EBM) Guidelines database with the intention to adapt them to the Belgian context [4]. This database contains (to date) 940 EBM Guidelines of Duodecim Medical Publications available in English.

The implementation of evidence-based information in a specific context is influenced by the interaction of determinants that can be grouped into 7 domains: guideline factors, individual health professional factors, patient factors, professional interactions, incentives and resources, capacity for organizational change, and social, political, and legal factors [5]. The accessibility of guidelines written in the mother tongue is an example of a specific factor that might influence an implementation strategy. Assuring the availability of international guidelines in the local language increases the chances that they are consulted by non-native English medical professionals and that the recommendations contained in them are better retained. Especially when guidelines are offered for point-of-care use by busy non-native English speaking physicians, translation in their mother language is essential for acceptance, ease of use, and adoption [6]. This point of view is based on limited but consistent evidence on the importance of language as a barrier to the use of evidence-based medicine [7-11]. The importance of translating English guidelines into Dutch and French was also pinpointed by the interviewees in a Belgian study on facilitating factors for the dissemination and implementation of guidelines [12]. It was for these reasons that the Belgian health care authorities ordered the translation of these Duodecim Guidelines from English into Dutch and French.

Considering the scale and potential cost of the translation project, it was decided to make use of computer-aided translation performed by certificated translators with limited expertise in medical translation. The output of the translations was subsequently revised by medical proofreaders (general practitioners).

The first cycle of 940 revised translations resulted in the construction of a validated medical domain-specific translation memory, possibly helpful for the translation of future new guidelines or future updates of existing guidelines. New Duodecim Guidelines, as well as updated existing guidelines, will continue to be translated using the same hybrid approach, supported by the translation memory. As this method involves human translation/validation as well as computer support, we will describe it as a “hybrid human and computer-assisted” approach. If the hybrid method were to approach the quality and speed of expert medical translation, the method may serve as a model to other major medical translation projects.

Therefore, this pilot study aims to compare the speed and quality of 3 approaches of translation: (1) a certificated junior translator without medical specialization using the hybrid method, (2) an experienced medical translator with medical specialization but without this support, and (3) a certificated junior translator without medical specialization without this support.

## Methods

### Construction of the Translation Memory

To construct a translation memory on the basis of the first 940 Duodecim Guidelines, junior translators (trained at the master’s level in general English-French and English-Dutch, but without special training in medical translations) used a hybrid approach, involving human translation supported by translation memory and by terminology recognition from terminology databases (termbases), as well as support from online machine translation. The software used was SDL Trados Studio.

The output of the translations was subsequently revised by medical proofreaders (general practitioners). The resulting corrected versions of the first 940 translations have now been converted to a validated medical domain-specific translation memory.

The principle of translation memory is that it stores source and target segments during human translation and offers translation suggestions on the basis of earlier translations when an identical or similar segment is submitted for translation. Approximately 75,000 translated segments were generated during the preparatory period. In addition, terminology recognition is used. Any term in a submitted segment that is also present in a termbase attached to the translation project is immediately marked and its equivalent term in the target language is displayed. The memory is stored in the sdltm format of SDL Trados Studio. It is compatible with the sdltb format of the SDL MultiTerm termbases, used earlier in the project (approximately 5000 terms and their translations). SDL Trados Studio allows immediate segment-specific access to Google Translate, which uses statistical methods to suggest translations based on large bilingual corpora.

Further details on the development of the translation memory and on the termbases are described elsewhere [1].

### Hybrid Method of Translation in the Experiment

In the experiment, junior translators used the same hybrid human and computer-aided approach, now relying on the full, validated translation memory as described earlier. The translators also made use of the termbases and Google Translate. The output was again validated by a medical proofreader.

The quality and speed of this method were compared with the quality and speed of 2 other translation methods based on human translation without the validated translation memory and without the termbases but allowing help from Google Translate.

### Research Question

The research question was “What is the efficiency, measured in terms of the quality of the translation output and the speed of the translation procedures, of translation by (1) a certificated junior translator without medical specialization using the hybrid method (arm A), (2) an experienced medical translator with medical specialization but without the support of the translation memory and the termbases (arm B), and (3) a certificated junior translator without medical specialization without the support of the translation memory and the termbases (arm C)?”

### Study Design

We conducted a three-armed study in which comparable guidelines were translated by the 3 described methods. Another approach could have been to translate the same guidelines by both A and B, but we decided not to do this because of costs.

We used stratified randomization according to the number of words and the Flesch Reading Ease formula to ensure that the 3 arms were as similar as possible [13]. The randomization was performed by a third person, who was not involved in this study. We used random number lists obtained from a randomization website [14]. Ethical committee approval was not required for this study.

### Source Guidelines

We drew our sample from the Duodecim Evidence-Based Medicine Guidelines collection. These guidelines are targeted at primary care, ambulatory care, and community hospitals. The Duodecim guidelines are available in English and present recommendations in a concise way to increase the usability at the point of care. The collection is updated on a continuous basis, and every 3 years the entire collection is revised. New guidelines are published regularly. From this collection, we included all 30 new guidelines published between August 2011 and January 2014. In addition, we selected 30 guidelines with major updates, correcting or amplifying earlier information.

### Hypothesis

The hypothesis was that the hybrid translation method, which allows reuse of earlier translations through its translation memory component, could substantially contribute to translation efficiency in the case of updated guidelines.

### Translation Procedures

**Figure 1:**
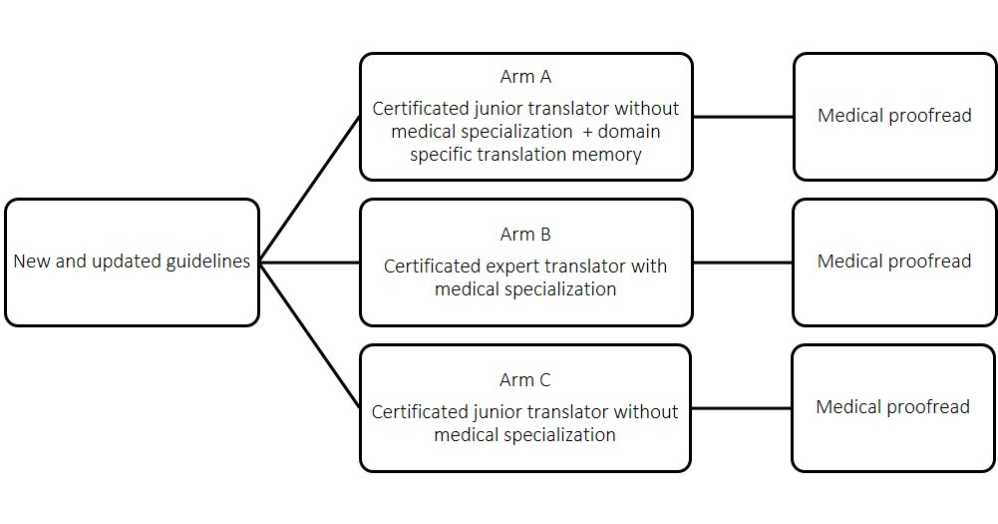
Translation procedures.

The procedures for each arm in the experiment are described in the following section and represented schematically in Figure 1.

In arm A, a certificated junior translator without medical specialization was asked to translate the English guidelines into Dutch using the hybrid approach explained earlier, that is, making use of the SDL Trados Studio translation software that provided input based on the translation memory (approximately 75,000 translated segments), the SDL MultiTerm termbases used earlier in the project (approximately 5000 terms and their translations), and support from Google Translate. The translator was also allowed to use other online and paper sources.

In arm B, an experienced medical translator (professional translator and editor at EBMPracticeNet) was allowed to use her own translation resources but did not have access to the validated translation memory or to the MultiTerm termbases. She was allowed to use input from Google Translate.

In arm C, the same certificated junior translator as in arm A translated guidelines to Dutch without access to the validated translation memory and to the MultiTerm termbases. The translator was allowed to use other sources, including Google Translate, that were deemed relevant but none were recommended to the translator by the team.

After translation, a medical proofreader, who was blinded to the translation procedure status, revised every translated guideline. Instructions for the medical proofreader included to repair nonsensical phrases; fix interpretation errors; rectify mistranslations, nontranslation, or inconsistent translation of terminology; and ensure that the text is understandable and stylistically acceptable to a Dutch native speaker who needs to understand the contents of the document [15]. The medical proofreader was also advised not to change text that is accurate and acceptable just for the sake of improving its style [16].

These procedures were applied once for the 30 new guidelines and once for 30 updated guidelines, but the junior translator was a different person in the second part of the experiment. By “certificated translator,” we mean a translator with a university or college degree in translation.

### Outcomes

#### Baseline Data

To evaluate the baseline comparability of the guidelines, for each guideline we counted the number of words and calculated the Flesch Reading Ease score [13]. The Flesch scale goes from 0 to 120. A lower score indicates more difficult text and a score below 30 is recommended only for a reader at the university graduate level. To calculate the score, we used Hendi, a tool developed to assess the readability of texts [17].

#### Translation Speed

Translation speed was measured by recording the translation time and the postediting time needed by the medical proofreader. For this purpose, we used Time Stamp [18]. To evaluate the speed of translation, we added up translation and revision time for each guideline.

#### Translation Quality

The output of the 3 types of translation was compared using the Human Translation Edit Rate (HTER). This is an automated metric based on edit distance that is usually used to calculate the minimum number of changes required for highly trained human editors to correct machine translation output so that it accurately reflects the meaning of the reference translation [19]. A higher HTER score indicates a higher number of changes. To calculate the HTER, we compared the output of every guideline after translation with the output after medical proofreading as the reference translation. The HTER was compared at the global text level.

**Figure 2:**
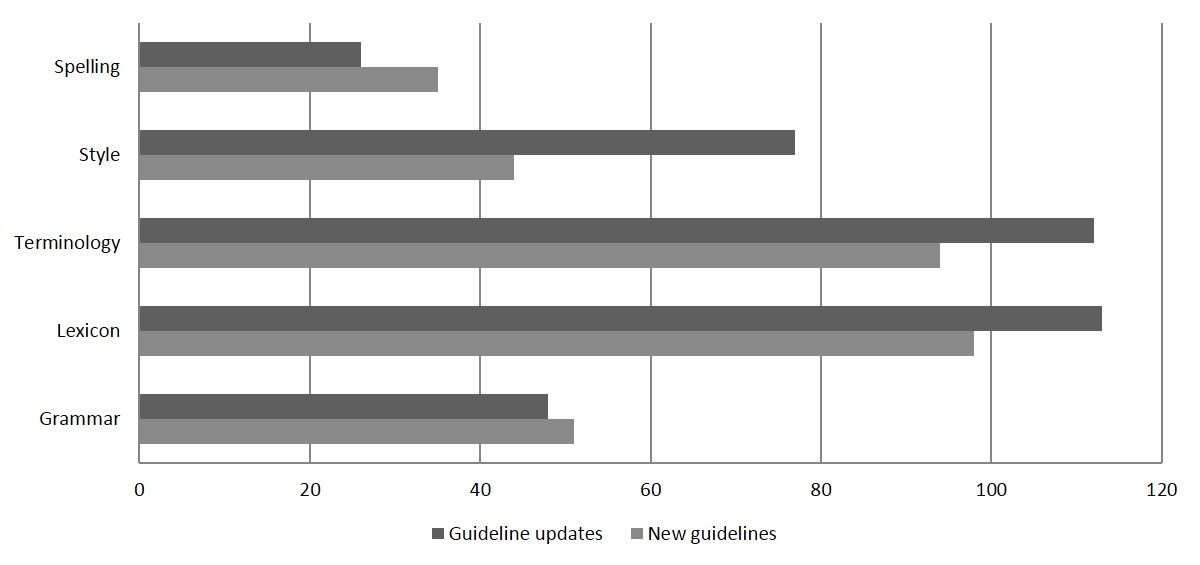
Number of revisions by the medical proofreader with respect to acceptability. Terminology refers to the use of other terms than those in the predefined list of preferred terms. Lexicon refers to bad word choice or use of wrong prepositions.

**Figure 3:**
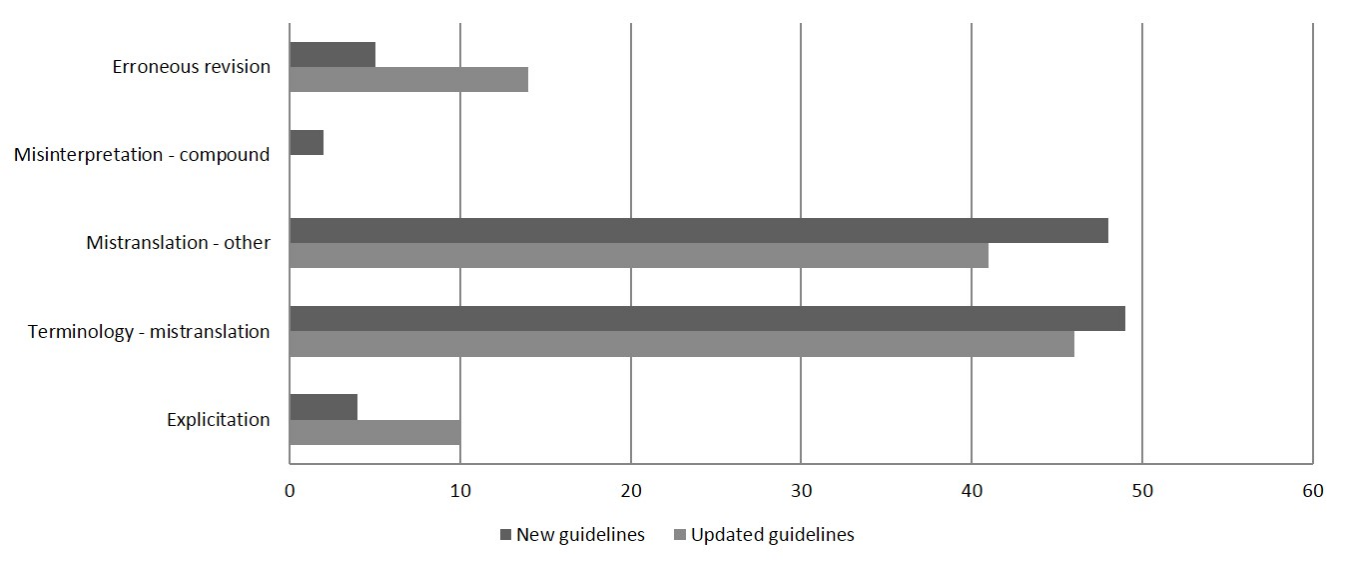
Number of revisions by the medical proofreader in relation to the adequacy domain. Explicitation means that the reviser amplified the translation to make its meaning more explicit. Terminology refers to the incorrect translation of terms, while Mistranslation refers to incorrect translation of other words. Misinterpretation indicates that a compound was misinterpreted.

To gain insights into the type of changes made by the medical proofreader, a further manual evaluation was made of translation acceptability and adequacy for 3 new and 3 updated guidelines per arm. Adequacy relates to the correspondence in meaning between source text and target text, whereas acceptability is the linguistic felicity of the target, that is, the use of suitable wording to express what was intended [20]. To evaluate adequacy and acceptability, we classified all revisions of the medical proofreader into 5 subcategories for adequacy and 5 subcategories for acceptability. See Figures 2 and 3 for an overview of the subcategories [21].

### Sample Size

Given that this was a pilot study with a fixed sample, we did not perform a priori sample size calculations.

### Statistical Methods

We used descriptive statistics to compare baseline characteristics (number of words and Flesch score) of the 3 groups. The time needed per 1000 words was calculated, and these ratios were compared between groups using a one-way analysis of variance (ANOVA) followed by Tukey honest significant difference (HSD) tests. A *P* value less than .05 was considered significant. Given that it is difficult to evaluate the normality assumption in small datasets, nonparametric tests were conducted as well to verify the robustness of the drawn conclusions (sensitivity analysis). More specifically, a Kruskal-Wallis test followed by pairwise Mann-Whitney U tests were performed.

## Results

### Baseline Data

The average number of words per guideline was 1195 (new guidelines=1172; updated guidelines=1218). This illustrates the concise character of the point-of-care guidelines. Because we selected updates with major revisions, the number of changed or new words is very high. The Flesch reading score ranged from 31.4 to 36.6 corresponding to readability at a university graduate level. Table 1 shows the baseline data per study arm for the 30 new guidelines. Data per study arm for the updated guidelines are presented in Table 2. The number of words per guideline varied from 210 to 4695, which explains the large standard deviations reported in the tables.

**Table 1:** Baseline data for the new guidelines per comparison group.

Arm	Baseline data, mean (SD)				
	Words	FRE score	Translator (Time/1000w)	Proofreader (Time/1000w)	Total (Time/1000w)
A^a^(n=10)	1252.4 (1334.8)	35.8 (10.3)	18.7 (4.1)	25.3 (9.8)	44.0 (12.2)
B^b^(n=10)	1320.2 (991.5)	32.3 (10.4)	51.7 (13.5)	14.8 (3.4)	66.5 (15.6)
C^c^(n=10)	943.8 (314.0)	36.1 (7.4)	22.6 (5.6)	23.4 (7.5)	45.9 (10.0)

^a^Certificated junior translator without medical specialization with domain-specific translation memory.

^b^Experienced medical translator without translation memory.

^c^Certificated junior translator without medical specialization without translation memory.

**Table 2:** Baseline data for the updated guidelines per comparison group.

Arm	Baseline data, mean (SD)					
	Words	Changed/new words	FRE score	Translator (Time/1000w)	Proofreader (Time/1000w)	Total (Time/1000w)
Arm A^a^(n=10)	1376.0 (1211.1)	945.9 (501.0)	31.4 (5.9)	130.0 (50.5)	22.9 (14.2)	151.7 (59.8)
Arm B^b^(n=10)	1100.0 (589.6)	1012.8 (642.0)	36.5 (5.5)	66.5 (20.6)	17.1 (8.3)	83.7 (27.1)
Arm C^c^(n=10)	1178.3 (637.4)	1070.7 (681.9)	36.6 (7.2)	192.8 (53.2)	17.6 (7.9)	210.4 (57.5)

^a^Certificated junior translator without medical specialization with domain specific translation memory.

^b^Experienced medical translator without translation memory.

^c^Certificated junior translator without medical specialization without translation memory.

### Translation Speed

Overall, the mean total translation time was 100.2 (70.7) minutes/1000 words. Translation by the experienced medical translator and medical proofreader took 66.5 min/1000 words for the new guidelines and 83.7 minutes/1000 words for the updated guidelines. Comparison of the updated and original text by the translator accounted for this difference. The time for translation by the junior translators was substantially different for the new versus updated guidelines. Because the junior translators were different persons for the new guidelines and the updated guidelines, these comparisons are not meaningful.

Translation of new guidelines by the junior translator and medical proofreader was 2 minutes/1000 words faster with support versus without, but this was not statistically significant (44 minutes/1000 words versus 46 minutes/1000 words; 95% CI -16 to 12; *P*=.94).

For the updated guidelines, the translation by the junior translator and medical proofreader was 59 minutes/1000 words faster with support versus without (152 minutes/1000 words versus 210 minutes/1000 words; 95% CI -115 to -2; *P*=.043 with parametric test and *P*=.053 with nonparametric test).

### Translation Quality

The experienced medical translator provided the best quality translations with an HTER score of 3.7 for the new guidelines and 4.2 for the updated guidelines. For the less experienced translators, we did not find any difference in quality in guidelines translated with or without computer assistance. In the test with the new guidelines, the HTER scores were 10.3 with support and 9.5 without. For the updated guidelines, the scores were 5.2 with support and 5.4 without.

On the basis of an analysis of 18 new and updated guidelines (3/arm), we identified 698 revisions by the medical proofreader that related to acceptability and 219 revisions in relation to adequacy. Further details are available in Figures 2 and 3. A third of the revisions were due to terminology. In-depth analysis of the number of terminological revisions per group did not demonstrate any relevant differences.

## Discussion

### Principal Findings

This study shows that the hybrid approach, that is, human and computer-aided translation, is useful when updates of clinical practice guidelines have to be translated. There was no apparent benefit for the translation of new guidelines. Use of the translation software did not increase the quality of the translations but significantly improved translation speed for updates of existing guidelines. This can be explained by the fact that the translation of unchanged or slightly changed segments is immediately suggested by the translation memory.

Speed of translation is important in the case of updated guidelines. When updating of guidelines is slow, there is an increased risk that guidelines will become out of date, which can affect quality of care. Median times to incorporate new evidence in updates of guidelines takes 10 months for the Duodecim EBM Guidelines [22]. Translating updated guidelines can increase the use of the guidelines but adds to the delay. It is therefore relevant to find that translation software can contribute to increasing efficiency in this particular case. We did not evaluate the outcomes for guidelines with minor updates, which represent the biggest part of the guideline collection. Our expectation is that here, too, the translation software will enhance efficiency.

The evaluation of adequacy and acceptability demonstrated that the performance of the translation procedures can be improved by the introduction of an automatic terminology consistency check. Terminology was the most important reason for revisions. Contrary to our expectations, the use of the MultiTerm termbases did not result in fewer terminological revisions. An explanation could be that the certificated translator without medical specialization delivered the same overall quality but needed more time to identify the relevant terms. Figure 2 also shows that although the medical proofreader was advised not to change text just for the sake of improving its style, a large number of stylistic changes were made. Providing style guides to the translators and proofreaders might make the process more efficient.

### Strengths and Limitations

A limitation of this study is that only 3 translators were involved, with substantial differences in working style. This made it difficult to make meaningful comparisons of the working speed between the different translators. While one of the junior translators worked faster than the experienced medical translator, this was counterbalanced by substantially lower quality scores and more time required for the medical proofread. Another limitation is that there was no monitoring of how the translators used the translation technology. Even though the translators were acquainted with the software, it was not established whether they made optimal use of it. Furthermore, the pilot study used a small guideline sample, and although care was taken to ensure comparability of the texts, this comparability was based on readability scores while arguably other factors may also play a role (eg, some subject matter is more difficult to grasp for the translator than others, regardless of readability scores). In light of these limitations, the results of this study should be interpreted with caution.

Another approach to translating these guidelines would have been to replace online machine translation by Google Translate with a dedicated machine translation component trained on selected bilingual medical data including the validated memory. A limitation of the translation memory approach is that it provides translation support only when there is a sufficient match value between a new sentence and one already stored in the memory. The machine translation approach, on the contrary, is able to combine partial matches into a new translation proposal (but in doing so may also offer more inadequate translation proposals). The Cochrane collaboration is currently using a machine translation approach to translate Cochrane reviews into several languages [23]. Epistemonikos, a multilingual database of the available health evidence, is another project that uses automated statistical machine translations [24]. The performance of our hybrid method supplemented with a dedicated machine translation system remains to be tested.

Because there is only limited evidence on the importance of translation to tackle language barriers, we believe it would be worthwhile to test the effect on reading speed and retention of information. Two previous studies illustrate how this can be tested with the design of a randomized controlled trial [7,8].

### Conclusions

The development and updating of guidelines is time consuming and expensive, and strategies are needed to increase cost effectiveness [25]. A large number of clinical practice guidelines and databases with evidence-based point-of-care information are available throughout the world. In a move toward more international collaboration, we expect that the exchange of high-quality guidelines between organizations internationally and the use of translation software can contribute to increasing the cost effectiveness of guidelines. This study provides preliminary evidence to support the usefulness of translation memory technology for keeping a translated set of guidelines up to date. Further research is needed to evaluate the usefulness of dedicated machine translation systems for the translation of new guidelines.

## Abbreviations

EBM: evidence-based medicine
HTER: Human Translation Edit Rate
Termbase: terminology database
